# Health and economic impact of improved glucose, blood pressure and lipid control among German adults with type 2 diabetes: a modelling study

**DOI:** 10.1007/s00125-023-05950-3

**Published:** 2023-06-30

**Authors:** Min Fan, Anna-Janina Stephan, Karl Emmert-Fees, Annette Peters, Michael Laxy

**Affiliations:** 1 Department of Sport and Health Sciences, Technical University of Munich, Munich, Germany; 2 German Center for Diabetes Research (DZD), Munich, Germany; 3 Institute of Health Economics and Health Care Management, Helmholtz Munich, Munich, Germany; 4 Institute of Epidemiology, Helmholtz Munich, Munich, Germany

**Keywords:** Diabetes, Long-term consequences, Risk factor control, Simulation modelling

## Abstract

**Aims/hypothesis:**

The aim of this study was to estimate the long-term health and economic consequences of improved risk factor control in German adults with type 2 diabetes.

**Methods:**

We used the UK Prospective Diabetes Study Outcomes Model 2 to project the patient-level health outcomes and healthcare costs of people with type 2 diabetes in Germany over 5, 10 and 30 years. We parameterised the model using the best available data on population characteristics, healthcare costs and health-related quality of life from German studies. The modelled scenarios were: (1) a permanent reduction of HbA_1c_ by 5.5 mmol/mol (0.5%), of systolic BP (SBP) by 10 mmHg, or of LDL-cholesterol by 0.26 mmol/l in all patients, and (2) achievement of guideline care recommendations for HbA_1c_ (≤53 mmol/mol [7%]), SBP (≤140 mmHg) or LDL-cholesterol (≤2.6 mmol/l) in patients who do not meet the recommendations. We calculated nationwide estimates using age- and sex-specific quality-adjusted life year (QALY) and cost estimates, type 2 diabetes prevalence and population size.

**Results:**

Over 10 years, a permanent reduction of HbA_1c_ by 5.5 mmol/mol (0.5%), SBP by 10 mmHg or LDL-cholesterol by 0.26 mmol/l led to per-person savings in healthcare expenditures of €121, €238 and €34, and 0.01, 0.02 and 0.015 QALYs gained, respectively. Achieving guideline care recommendations for HbA_1c_, SBP or LDL-cholesterol could reduce healthcare expenditure by €451, €507 and €327 and gained 0.03, 0.05 and 0.06 additional QALYs in individuals who did not meet the recommendations. Nationally, achieving guideline care recommendations for HbA_1c_, SBP and LDL-cholesterol could reduce healthcare costs by over €1.9 billion.

**Conclusions/interpretation:**

Sustained improvements in HbA_1c_, SBP and LDL-cholesterol control among diabetes patients in Germany can lead to substantial health benefits and reduce healthcare expenditures.

**Graphical Abstract:**

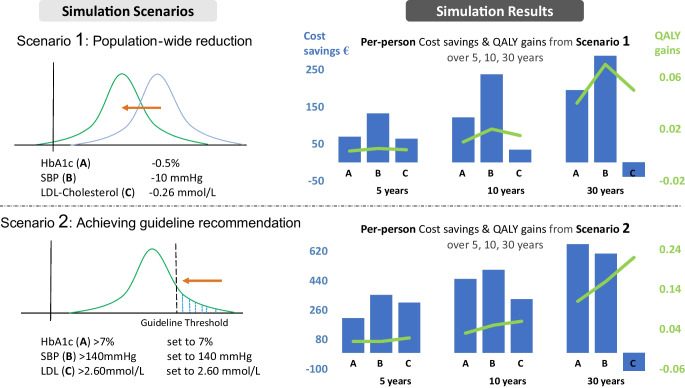

**Supplementary Information:**

The online version contains peer-reviewed but unedited supplementary material available at 10.1007/s00125-023-05950-3.


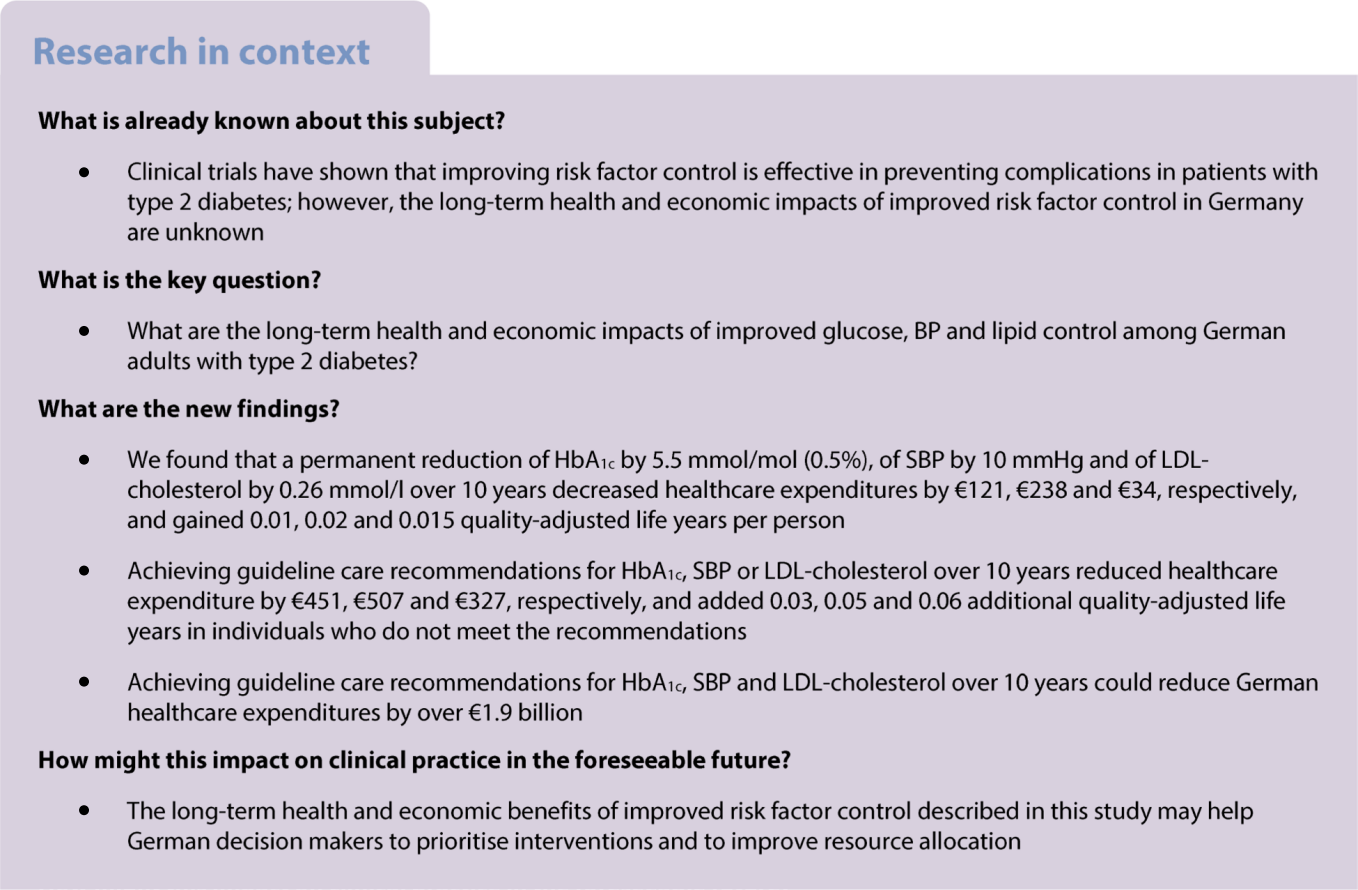


## Introduction

As of 2010, over 7% of the German adult population had a type 2 diabetes diagnosis [1], and its prevalence is expected to increase substantially over the next two decades [2]. The costs for routine diabetes management, care for diabetes complications, the time required for self-management and diabetes-associated productivity losses place a substantial economic and health burden on patients, healthcare systems and societies [3–6]. Previous studies have shown that lifestyle and pharmaceutical interventions aiming at better risk factor control are effective in delaying and preventing subsequent complications and death [7–9]. Despite this robust evidence, achievement of guideline-recommended risk factor levels remains sub-optimal globally [10]. In Germany, national surveys have suggested that approximately one-third of patients with type 2 diabetes failed to achieve the guideline-recommended targets for HbA_1c_ and BP, and over 70% did not achieve the guideline care recommendation for LDL-cholesterol [11, 12].

Quantifying the net health and economic burden of poor risk factor control is challenging. Due to the slow progression of type 2 diabetes, the benefits of improved risk factor control accumulate over years or even decades [7, 13, 14], while implementation of interventions to improve risk factor control usually requires considerable up-front investment. Evaluating the long-term value of improved risk factor control is therefore crucial for decision-making given scarce resources in healthcare systems. Computer simulation models that predict long-term health outcomes of people with type 2 diabetes depending on their risk profiles have been established as useful tools that can help to fill this knowledge gap [15]. Previous studies from the US and UK that employed a modelling approach indicated that better risk factor control can lead to substantial reductions in healthcare costs and prolongation of quality-adjusted life expectancy among patients with type 2 diabetes [16–18]. However, as the underlying characteristics of patients and healthcare systems differ between countries, the results of these studies are highly context-specific, and the health and economic implications of improved risk factor control in Germany are unknown.

The objective of this study was to estimate the long-term per-person and nationwide health benefits and cost savings that could be achieved through improved control of HbA_1c_, systolic Blood pressure and LDL-Cholesterol (ABC control) in German adults with type 2 diabetes using a simulation modelling approach.

## Methods

### Overall study design

For the simulations, we used the UK Prospective Diabetes Study Outcome Model 2 (UKPDS-OM2), which can predict the development of diabetes-related complications, and estimates long-term patient-level health and economic outcomes [19]. We parameterised the model using patient characteristics from a large population-based German cohort study and utility decrements and healthcare expenditures associated with diabetes complications estimated from German survey and claims data [3, 4]. We simulated the mean per-person long-term health and economic consequences of two hypothetical improvement scenarios in ABC control for people with type 2 diabetes in Germany over 5, 10 and 30 years from a healthcare system perspective for the reference year 2022. To obtain estimates at the population level for Germany, we calculated person-level results stratified by age and sex, and combined them with the estimated number of adults with type 2 diabetes by age and sex in Germany.

### Intervention scenarios of improved ABC control

In the first scenario, we shifted the entire population distribution of ABC levels to the left (i.e. risk reduction). In the second scenario, we only shifted ABC levels of individuals whose levels would otherwise exceed the thresholds of guideline care recommendations. For each scenario, we estimated the health and economic effects of improved control in HbA_1c_, systolic blood pressure (SBP) or LDL-cholesterol separately. In addition, we simulated a ‘combined’ intervention for each scenario, in which all three risk factors were targeted simultaneuosly.

The improvements of ABC control in all the scenarios were assumed to be permanent over the respective simulation time horizon.

#### Population-level shift of ABC

We simulated the effects of a population-wide shift of the HbA_1c_ distribution by 5.5 mmol/mol (0.5%) [20], of the SBP distribution by 10 mmHg [21], and of the LDL-cholesterol distribution by 0.26 mmol/l [22]. This change in risk factor levels was irrespective of whether the original level was below or above the guideline-recommended threshold. The chosen effect size for the change in risk factors corresponds to around 20–50% of the standard deviations for the risk factors. These simulated intervention effects are higher than the effects resulting from lifestyle interventions [23], but lower than the effects resulting from intense pharmaceutical treatment [24–26], and therefore represent realistic changes of what could be achieved across the treatment cascade.

#### ABC guideline care recommendations

In this scenario, we only targeted individuals with ABC levels above the recommended thresholds mentioned in German or international guidelines. Acknowledging that the thresholds differ between countries and change over time and due to personalisation will not be applicable for each patient, we programmatically set them in this study to HbA_1c_ ≤53 mmol/mol (7%) for glycaemic control, SBP ≤140 mmHg for blood pressure management and LDL-cholesterol ≤2.6 mmol/l for lipid management. Individuals with risk factors above the threshold were set to the threshold level. For example, if the HbA_1c_ level of a person was 65 mmol/mol (8.1%) (above the guideline threshold), it was set to 53 mmol/mol (7%), whereas a person with an HbA_1c_ of 49.7 mmol/mol (6.7%) (below the guideline threshold) was assumed to be unaffected by the intervention.

#### Cost associated with changes in risk factor control

There are multiple ways of improving ABC control. These include intensified medication, better self-management, lifestyle changes or population-level intervention. All of these interventions come at a certain cost [27]. However, this study only aims to quantify the hypothetical population impact of improved ABC control independently of the measure used to achieve this improvement. Therefore, we disregarded the potential costs that any intervention would entail. Thus, the results of this study must always be considered in the context of the one-off or continuous cost of the intervention needed to improve ABC control.

### Simulation model

#### General information on the UKPDS-OM2

The UKPDS-OM2 is a patient-level microsimulation tool for modelling the progression of type 2 diabetes and estimating lifetime health outcomes of people with type 2 diabetes [19]. It was developed based on 89,760 patient-years of data from the UK Prospective Diabetes Study, a randomised controlled trial evaluating treatment regimens with different intensities of blood glucose and BP control, performed among newly diagnosed type 2 diabetes patients between 1977 and 1991 in the UK, and a 10-year post-trial monitoring observational study. Details on the development and validation of the model have been described in detail elsewhere [19, 28] and are briefly summarised here.

Risk equations in the UKPDS-OM2 were developed via proportional hazards survival models for first occurrence of eight diabetes complications (myocardial infarction, ischaemic heart disease, stroke, congestive heart failure, amputation, renal failure, blindness and foot ulcer), three additional second events (myocardial infarction, stroke, amputation) and mortality [19]. The probability of death and experiencing one or more complications is calculated for each patient at an annual cycle. As the model is parameterised with the respective utility decrements and healthcare expenditures associated with each complication, it predicts total accumulated healthcare costs and (quality-adjusted) life expectancy [28].

In a previous study, we tested the performance of the epidemiological risk equations in the UKPDS-OM2 by predicting mortality and cardiovascular events in a population-based German sample of people with type 2 diabetes. The results suggested that the risk equations overestimate mortality but predict cardiovascular events well over a 10-year time horizon, and that, despite some limitations, the model may be suitable for applications in German populations [29].

#### Model parameterisation with type 2 diabetes patient characteristics

To parameterise the model with patient characteristics that represent the mean German population with type 2 diabetes, we used data from participants with known type 2 diabetes (*n*=146) from the KORA-S4 study, a population-based cross-sectional survey and examination study of 4261 participants that was performed between 1999 and 2001 in the city of Augsburg and two surrounding counties in southern Germany. The study collected demographic and behavioural characteristics (age, sex, duration of diabetes, weight, height, smoking), biological risk factors (HDL-cholesterol, LDL-cholesterol, HbA_1c_, SBP, heart rate, white blood cell count, haemoglobin level and eGFR), and the occurrence of diabetes-related pre-existing events (ischaemic heart disease, heart failure, myocardial infarction, stroke, amputation, blindness, renal failure, ulcer) and the time that had passed since their occurrence, and some additional conditions (peripheral vascular disease, atrial fibrillation, albuminuria). We assumed that all patients were white and of European descent. We further adjusted the mean HbA_1c_, SBP and LDL-cholesterol levels in this sample for observed improvements that occurred between the years 2000 and 2014 [11]. To avoid instability in our simulations, we generated a synthetic population of 1000 patients based on the KORA-S4 sample using the R package ‘Synthpop’ that draws on the underlying empirical risk factor distributions (see electronic supplementary material [ESM] Appendix A for further details). This synthetic population was used to populate the model.

#### Model parameterisation with healthcare expenditures estimates

We used published estimates of total healthcare expenditures (including inpatient and outpatient care, pharmaceuticals, rehabilitation, and non-medical aids and remedies) associated with incident complications in patients with type 2 diabetes [3]. The study reported age- and sex-specific estimates of annual healthcare expenditures from the perspective of the German statutory health insurance system for the year of the event and in subsequent years in people with type 2 diabetes for all eight complications simulated in the UKPDS-OM2 (for further details, see ESM Appendix B and ESM Table 1). Costs are expressed in 2022 euros using official inflation data from the Statistisches Bundesamt (Federal Statistical Office) [30].

#### Model parameterisation with utility decrement estimates

Estimates on health utility decrements associated with type 2 diabetes and diabetes-related complications in Germany were taken from the KORA Health Questionnaire 2016, in which health utility was assessed using the EuroQol five-dimension questionnaire (EQ-5D-5L) in a sample of 8951 participants [4]. Since the utility decrements for ‘amputation’ and ‘renal failure’ were not estimated in this study, values for these two variables were taken from international studies [31, 32] (see ESM Appendix C and ESM Tables 2 and 3 for details).

#### Model details

Simulations were run over 5, 10 and 30 years; 30 years is assumed to approximate a population lifetime horizon. Healthcare expenditures and quality-adjusted life years (QALYs) were discounted by 3.5% per year. The number of internal loops (Monte-Carlo trials, related to first-order uncertainty; a sufficient number of loops can reduce the random variation of predicted outcomes in each internal loop) was set to 10,000, and the number of bootstrap samples was set to 100 (related to second order uncertainty; each bootstrap run will use a different set of model equation parameters from the 5000 sets of model equation parameters in OM2) to generate sufficiently precise confidence intervals around predicted outcomes.

### Outcomes

#### Costs and quality-adjusted life years

The cumulative healthcare costs and cumulative QALYs over simulation years were estimated.

#### Individual-level perspective

In the analysis of scenario with a population-level shift of the ABC distribution, we estimated mean per-person health and cost outcomes. In the analysis of the ABC guideline care recommendation scenario, we estimated mean per-person health and cost outcomes only for those above the recommended guideline threshold. We also generated mean per-person estimates representative for the German population by adjusting for the age and sex distribution of type 2 diabetes patients in Germany.

#### National healthcare system perspective

We extrapolated the modelled per-person effects to the whole German population with type 2 diabetes. Given that the patient characteristics from the KORA-S4 sample may not be fully representative of people with type 2 diabetes in the overall German population with type 2 diabetes, and that health outcomes are largely determined by age and sex, we multiplied the age- and sex-specific per-person effects (presented in ESM Appendix D) by the age- and sex-specific numbers of people with type 2 diabetes in Germany [1, 33]. Methodological details are given in ESM Appendix E.

For the guideline care scenario, we multiplied the age- and sex-specific per-person effects for those above the guideline care recommendation thresholds (presented in ESM Appendix D) by the age- and sex-specific numbers of people with type 2 diabetes in Germany and the age- and sex-specific proportions of people with type 2 diabetes whose ABC levels exceed recommended thresholds (age- and sex-specific prevalence is presented in ESM Table 9). We took these proportion data from our KORA-S4 sample, as the proportions of people with type 2 diabetes whose ABC levels exceed recommended thresholds in KORA lie in the middle of the reported proportions for other studies [10–12].

#### Maximum intervention costs at a given willingness-to-pay threshold

Based on the individual-level results for QALY gains and cost savings, we calculated the maximum costs for an intervention achieving the modelled risk factor improvements under which this intervention would be cost-effective over a lifetime, given a willingness-to-pay threshold of €50,000/QALY gained. We divided this number by the mean simulated life expectancy to annualise the maximum per-person expenditures.

### Sensitivity analyses

Several sensitivity analyses were performed to determine the relative importance of model parameters. We varied discount rates (0%, 5%), the number of internal loops (5000, 20,000), cost estimates and utility decrements (half of the original values, twice the original values).

## Results

### Sample characteristics

The baseline characteristics of participants with type 2 diabetes in the original KORA-S4 sample and the derived synthetic population are presented in Table 1. The mean age in the sample population was 62 years, the mean duration of diabetes was 9 years, the mean HbA_1c_ levels was 50.8 mmol/mol (6.8%), the mean SBP was 126 mmHg and the mean LDL-cholesterol was 2.93 mmol/l. The derived synthetic population had very similar characteristics.

**Table 1:** Baseline characteristics of the KORA-S4 sample and the synthetic model population

	KORA-S4 (*N*=146)	Synthetic population (*N*=1000)
Age (years)	62.1±8.9	61.9±9.0
Duration of diabetes (years)	9±8	9±7
BMI (kg/m^2^)	31.9±5.7	32.0±5.8
HbA_1c_ (mmol/mol)^a^	50.8±15.3	50.8±15.3
HbA_1c_ (%)^a^	6.8±1.4	6.8±1.4
SBP (mmHg)^a^	126±20.6	126±20.2
LDL-cholesterol (mmol/l)^a^	2.93±1.1	2.96±1.1
HDL-cholesterol (mmol/l)	1.23±0.33	1.21±0.32
% above HbA_1c_ GLC (53 mmol/mol)	34	34
% above SBP GLC (140 mmHg)	21	20
% above LDL-cholesterol GLC (2.6 mmol/l)	60	61
% above ABC GLC^b^	79	79
Ethnicity (white)^c^	146 (100)	1000 (100)
Sex (male)	78 (53)	539 (54)
Smoker (yes)	21 (14)	143 (14)
History of disease (%)		
Ischaemic heart disease	9	9
Heart failure	14	16
Myocardial infarction	16	16
Stroke	6	7
Amputation	5	4
Blindness	1	1
Renal failure	3	3
Foot ulcer	0	0

Values for continuous variables are means ± SD; values for categorical variables are *n* (%)

^a^The mean values for HbA_1c_, SBP and LDL-cholesterol for the KORA-S4 sample are adjusted values, based on the adjusted differences between 2000 and 2014

^b^These values represent the percentage of people whose level for at least one risk factor was above the guideline-recommended threshold

^c^We assumed that all patients were white and of European descent

GLC, guideline care recommendation

### Per-person cost savings and QALY gains

Differences in total healthcare costs and QALYs between the intervention scenarios and the control scenario without any risk factor improvements are driven by reduced event rates of complications in the group with improved ABC control. For example, a reduction of systolic BP of 10 mmHg over a time horizon of 10 years reduced the cumulative incidence of stroke from 5% to 4.4%. The differences in cumulative event rates of the eight modelled complications between reference and treatment groups over the various simulation time horizons are reported in ESM Appendix F. Improved HbA_1c_ control mainly had an impact on microvascular outcomes (foot ulcer, blindness or amputation), whereas the effect of improvements in LDL-cholesterol was mostly limited to cardiovascular events (stroke, myocardial infarction and ischaemic heart disease).

Table 2 shows the per-person total healthcare expenditure, QALYs and remaining life years for the reference group, and the incremental healthcare expenditures (cost savings; negative cost savings represent an increase in healthcare expenditures), QALYs and life expectancies between the reference group and each intervention group. The simulated mean per-person estimates are based on the characteristics of type 2 diabetes patients in the KORA-S4 sample.

**Table 2:** Per-person impact of improvements and adherence to German guideline care recommendations for HbA_1c_, SBP and LDL-cholesterol over 5, 10 and 30 years

	5 years			10 years			30 years		
	∆Cost	∆QALYs	∆LYs	∆Cost	∆QALYs	∆LYs	∆Cost	∆QALYs	∆LYs
HbA_1c_									
Reduction of 5.5 mmol/mol (0.5%)	69 (43, 92)	0.003 (0.002, 0.004)	0.003 (0.002, 0.004)	121 (83, 182)	0.01 (0.007, 0.013)	0.01 (0.007, 0.013)	195 (80, 293)	0.04 (0.03, 0.05)	0.04 (0.03, 0.05)
Guideline care (≤53 mmol/mol [≤7%])	211 (154, 297)	0.009 (0.005, 0.011)	0.009 (0.004, 0.012)	451 (344, 676)	0.03 (0.02, 0.04)	0.03 (0.02, 0.04)	664 (518, 1138)	0.11 (0.08, 0.14)	0.12 (0.09, 0.15)
SBP									
Reduction of 10 mmHg	132 (90, 167)	0.005 (0.004, 0.007)	0.005 (0.003, 0.007)	238 (154, 307)	0.02 (0.014, 0.023)	0.02 (0.01, 0.03)	288 (128, 408)	0.07 (0.05, 0.09)	0.08 (0.06, 0.1)
Guideline care (≤140 mmHg)	353 (234, 470)	0.009 (0.008, 0.018)	0.008 (0.007, 0.019)	507 (349, 922)	0.05 (0.03, 0.06)	0.05 (0.03, 0.07)	605 (179, 1154)	0.16 (0.12, 0.20)	0.18 (0.13, 0.23)
LDL-cholesterol									
Reduction of 0.26 mmol/l	64 (29, 90)	0.004 (0.003, 0.005)	0.005 (0.003, 0.006)	34 (29, 115)	0.015 (0.011, 0.018)	0.018 (0.013, 0.022)	−39 (−115, 29)	0.05 (0.04, 0.06)	0.06 (0.05, 0.08)
Guideline care (≤2.60 mmol/l)	306 (203, 396)	0.018 (0.015, 0.024)	0.021 (0.017, 0.028)	327 (230, 515)	0.06 (0.05, 0.08)	0.08 (0.06, 0.10)	−111 (−352, 141)	0.22 (0.18, 0.28)	0.27 (0.22, 0.35)
Combined population-wide reduction	254 (202, 294)	0.012 (0.010, 0.014)	0.012 (0.011, 0.015)	422 (339, 514)	0.04 (0.04, 0.05)	0.05 (0.04, 0.05)	438 (190, 589)	0.15 (0.14, 0.17)	0.17 (0.16, 0.20)
Combined guideline care recommendations^a^	400 (316, 477)	0.020 (0.018, 0.024)	0.022 (0.019, 0.027)	581 (462, 776)	0.07 (0.06, 0.08)	0.08 (0.07, 0.10)	451 (170, 695)	0.25 (0.22, 0.29)	0.29 (0.26, 0.35)

Values are presented as mean (95% CI)

ΔCost represents per-person savings in healthcare expenditures (negative values represent an increase in healthcare expenditures) achieved through the intervention scenarios; ΔQALYs represent per-person QALYs gained through the intervention scenarios; ΔLYs represent per-person life years gained through the intervention scenarios

Mean per-person accumulated healthcare expenditures, QALYs and LYs without the intervention (reference) for people with type 2 diabetes in Germany were €30,987, 3.2 QALYs and 4.1 LYs over 5 years,  €51,821, 5.5 QALYs and 6.9 LYs over 10 years, and €80,528, 8.7 QALYs and 11 LYs over 30 years

^a^The ‘combined guideline care recommendations’ scenarios were for those patients who did not meet at least one of the guideline-recommended thresholds for the risk factors; their above-threshold risk factor level was set to the threshold level

Sustained population-wide decreases of HbA_1c_ by 5.5 mmol/mol (0.5%) (mean 50.8 mmol/mol [6.8%] vs 45.3 mmol/mol [6.3%]), of SBP by 10 mmHg (mean SBP of 126 mmHg vs 116 mmHg) and of LDL-cholesterol by 0.26 mmol/l (mean 2.96 mmol/l vs 2.70 mmol/l) over 10 years led to gains in QALYs of 0.01 (95% CI 0.007, 0.013), 0.02 (0.014, 0.023) and 0.015 (0.011, 0.018), respectively, and healthcare cost savings of €121 (€83, €182), €238 (€154, €307) and €34 (€29, €115) per person, respectively. Achieving guideline care recommendations for HbA_1c_, SBP and LDL-cholesterol over 10 years resulted in QALYs gained of 0.03 (0.02, 0.04), 0.05 (0.03, 0.06) and 0.06 (0.05, 0.08), respectively, and decreased healthcare costs by €451 (€344, €676), €507 (€349, €922) and €327 (€230, €515), respectively, per person with ABC levels above the recommended threshold. Generally, QALY gains and healthcare savings were substantially smaller for the 5-year time horizon and substantially larger for the 30-year time horizon, with the exception that healthcare cost savings were smaller and negative for LDL-cholesterol control over the 30-year time horizon, i.e. the costs increased.

Simultaneously decreasing HbA_1c_ by 5.5 mmol/mol (0.5%), SBP by 10 mmHg and LDL-cholesterol level by 0.26 mmol/l (‘Combined population-wide reduction’ in Table 2) led to a per-person QALY gain of 0.012 (0.010, 0.014) over 5 years, of 0.04 (0.04, 0.05) over 10 years and of 0.15 (0.14, 0.17) over 30 years (lifetime), and reduced per-person healthcare costs by €254 (€202, €294), €422 (€339, €514) and €438 (€190, €589), respectively. Achieving guideline care recommendations for HbA_1c_, SBP and LDL-cholesterol resulted in QALY gains of 0.02 (0.018, 0.024), 0.07 (0.06, 0.08) and 0.25 (0.22, 0.29) over 5, 10 and 30 years, respectively, and healthcare cost savings of €400 (€316, €477), €581 (€462, €776) and €451 (€170, €695) per person for people with at least one value above the ABC thresholds.

ESM Table 10 shows the per-person estimates after adjusting for the age and sex distribution of patients with type 2 diabetes in Germany. The per-person QALY and life years gained for the German population are very similar to those in Table 2; per-person cost savings are slightly higher than the estimates from the KORA-S4 sample over the 5-year time horizon, and slightly lower compared with those from the KORA-S4 sample over the 30-year time horizon.

### Cost savings and QALY gains from a national healthcare system perspective

The nationwide cost savings and QALY gains resulting from improved ABC control from a national healthcare system perspective are shown in Table 3. Over a 10-year period, sustaining a population-wide reduction of HbA_1c_ by 5.5 mmol/mol (0.5%), of SBP by 10 mmHg and of LDL-cholesterol by  0.26 mmol/l would result in 54,381, 98,753 and 78,535 QALYs gained, respectively, and €543 million, €1047 million and €60 million cost savings, respectively, for people with type 2 diabetes in Germany; achieving recommended levels of HbA_1c_, SBP and LDL-cholesterol for every patient with type 2 diabetes in Germany would lead to 43,358, 45,418 and 185,381 QALYs gained, respectively, and reduce healthcare expenditures by €681 million, €457 million and €921 million, respectively.

**Table 3:** Population-level impact of improvements and adherence to German guideline care recommendations for HbA_1c_, SBP and LDL-cholesterol over 5, 10 and 30 years

	5 years			10 years			30 years		
	ΔCost (M)	ΔQALYs	ΔLYs	ΔCost (M)	ΔQALYs	ΔLYs	ΔCost (M)	ΔQALYs	ΔLYs
HbA_1c_									
Reduction of 5.5 mmol/mol (0.5%)	377	14,528	12,546	543	54,381	55,886	805	162,388	171,954
Guideline care (≤53 mmol/mol [≤7%])	390	12,688	10,844	681	43,358	43,459	863	148,935	159,887
SBP									
Reduction of 10 mmHg	650	23,726	23,649	1047	98,753	108,448	1323	294,926	336,083
Guideline care (≤140 mmHg)	342	6690	5537	457	45,418	49,569	510	118,519	134,369
LDL-cholesterol									
Reduction of 0.26 mmol/l	313	18,166	21,036	60	78,535	96,594	−156	248,063	267,651
Guideline care (≤2.60 mmol/l)	961	49,269	56,358	921	185,381	223,004	−237	575,926	706,120
Combined population-wide reduction	1267	58,659	60,646	1808	210,333	232,231	1848	661,514	763,368
Combined guideline care recommendations^a^	1443	74,209	81,297	1904	268,783	309,869	1287	828,692	980,049

Values describe population-level estimates for approximately 4.6 million people with type 2 diabetes in Germany

ΔCost represent population-level savings in healthcare expenditures (negative values represent an increase in healthcare expenditures) achieved through the intervention scenarios; ΔQALYs represent population-level QALYs gained through the intervention scenarios; ΔLYs represent population-level life years gained through the intervention scenarios

The accumulated healthcare expenditures, QALYs and LYs at population level without the intervention (reference) for people with type 2 diabetes in Germany were €146,188 million, 14 million QALYs and 18 million LYs over 5 years, €234,499 million, 24 million QALYs and 30 million LYs over 10 years, and €341,764 million, 36 million QALYs and 46 million LYs over 30 years

^a^The ‘combined guideline care recommendations’ scenarios were for those patients who did not meet at least one of the guideline-recommended thresholds for the risk factors; their above-threshold risk factor level was set to the threshold level

M, millions

Shifting the ABC risk factor distribution simultaneously for all type 2 diabetes patients in Germany would gain 210,333 QALYs and reduce healthcare costs by over €1.8 billion within the national healthcare system over 10 years. Achieving recommended levels of ABC control for everyone with levels above the threshold would lead to a decrease of over €1.9 billion in healthcare expenditure, and an increase of 268,783 QALYs.

### Maximum per-person annual intervention costs at a willingness-to-pay threshold of €50,000 per QALY

To be cost-effective at a willingness-to-pay threshold of €50,000 per QALY gained over a lifetime perspective, we found that the annual cost for a hypothetical intervention to achieve guideline care recommendations for HbA_1c_, SBP or LDL-cholesterol should not exceed €549, €760 or €958, respectively (Table 4).

**Table 4:** Maximum per-person annual costs of interventions for them to be cost-effective with a willingness-to-pay threshold of €50,000 per QALY gained over a 30-year time horizon (lifetime)

Intervention outcome	Maximum per-person annual intervention cost (€)^a^
HbA_1c_	
Reduction of 5.5 mmol/mol (0.5%)	190
Guideline care (≤53 mmol/mol [≤7%])	549
SBP	
Reduction of 10 mmHg	331
Guideline care (≤140 mmHg)	760
LDL-cholesterol	
Reduction of 0.26 mmol/l	230
Guideline care (≤2.60 mmol/l)	958
Combined population-wide reduction	712
Combined guideline care recommendations^b^	1139

^a^Maximum per-person annual intervention cost = [(willingness-to-pay threshold × QALYs gained) + cost saving]/years lived

^b^The ‘combined guideline care recommendations’ scenarios were for those patients who did not meet at least one of the guideline-recommended thresholds for the risk factors; their above-threshold risk factor level was set to the threshold level, i.e. in this scenario patients could be treated for one, two or three risk factors

### Sensitivity analyses

The Tornado plots in ESM Appendix G show the results of a one-way sensitivity analysis of simulated outcomes. Assumptions regarding the costs associated with complications, utility decrements for complications and the chosen discount rates had a bigger impact on the outcomes than the chosen number of loops.

## Discussion

### Summary of results

To the best of our knowledge, this is the first study to use an established, validated simulation model to predict long-term health and economic consequences of improved ABC control in German adults with type 2 diabetes. Our study showed that simultaneously achieving the recommended levels for HbA_1c_, SBP or LDL-cholesterol control for all people with type 2 diabetes in Germany would gain approximately 270,000 QALYs and save over €1.9 billion in healthcare costs over the next 10 years. The expected health benefits would be substantially greater over longer time horizons.

The two modelled scenarios – population-level shift of ABC and achievement of ABC guideline care recommendations – may be achieved by various evidence-based intervention strategies. A population-level shift of the risk factor distribution to the left may most likely be reached through a ‘population-level’ approach. An example for this is a national salt reduction intervention that would reduce salt intake in the entire population [34, 35] or the use of drugs for primary prevention [36]. In contrast, a scenario that only targets individuals who do not achieve guideline-recommended risk factor levels may be reached through a ‘high-risk screen and treat’ approach that involves pharmaceutical treatment, for example [34, 37, 38].

The magnitude of the healthcare cost savings and QALY gains at the national level are often greater for the scenarios in which the ABC distribution was shifted, even though individual-level effects were larger for the achievement of guideline care recommendations. This is because the achievement of guideline care recommendation scenarios only affected a proportion of patients—those with risk factor levels above the thresholds. For example, over 10 years, the healthcare cost savings per person were €238 for a 10 mmHg shift in the SBP distribution and €507 per person who achieved guideline-recommended levels for those whose SBP levels were above the threshold of 140 mmHg at baseline. As only 20% of the population had SBP levels above 140 mmHg, the national-level cost savings for the distribution shift were much larger (€1047 million) than those for achieving recommended levels of SBP (€457 million). This highlights the importance of complementing clinical disease management with population-based intervention strategies [34].

Achieving recommended levels for ABC control would reduce the healthcare expenditures of adults with diabetes by around €289 million annually (€1443 million saved over 5 years). In relative terms, this would reduce annual healthcare expenditures for patients with diabetes in Germany by approximately 1% [39].

### Results in light of the existing literature

Using the original model parameters from the UKPDS-OM2, Hayes et al reported that a decrease of 1.5% (16.4 mmol/mol) in HbA_1c_ led to an increase of 0.35 life years over lifetime [19]. This effect is larger compared with that observed in our study (0.04 life years with a 0.5% [5.5 mmol/mol] reduction in HbA_1c_), even after taking into account the intervention intensity. However, in their analysis, the study population had a worse baseline mean HbA_1c_ level of 8.2% (66.1 mmol/mol), compared with our 6.8% (50.8 mmol/mol), and the mean age of their study sample at baseline was 53 years (62 years in our study), which resulted in an expected life expectancy of 19.5 years in their study, almost twice as high as that in ours (11 years [Table 2]) [40].

The effects of improved ABC control on QALYs were also estimated using observed trial data. In the original UKPDS trial, a mean decrease in HbA_1c_ of 0.9% (9.8 mmol/mol) (from 7.9% [62.8 mmol/mol] to 7% [53.0 mmol/mol]) resulted in 0.08 QALYs gained over a time horizon of 10 years, and a mean decrease in SBP of 10 mmHg resulted in 0.1 QALYs gained [21, 41]. In comparison, in our study, 11 mmol/mol (1.0%) decrease in HbA_1c_ sustained over 10 years resulted in 0.02 QALYs gained, and a sustained decrease of SBP by 10 mmHg (from 154 to 144 mmHg) resulted in 0.02 QALYs gained (Table 2 and ESM Table 14). Therefore, our modelled QALYs gained were smaller compared with the UKPDS trial and predicted model scenarios in other populations.

Other previous modelling studies also reported higher QALY gains and cost savings from treatment scenarios that improve ABC control than those found in our study. A study by Palmer et al using the CORE Diabetes Model suggested that a decrease in HbA_1c_ of 0.9% (9.8 mmol/mol) would lead to gains of 0.81 QALYs and healthcare expenditure savings of US$10,800 per person over their lifetime from a US private payer perspective [16]. These differences may partially be explained by differences in the characteristics of the baseline population, the costs associated with treatment of diabetes and its complications, and the risk equations used to describe the epidemiological relationship between risk factor control and complications. In this context, it should be highlighted that, in contrast to this study [16] and other previous modelling studies, the performance of the risk equations used in our model was tested previously in the population of interest, showing that the calibration for cardiovascular morbidity and mortality is moderate to good [29]. In addition, the parameters for the costs associated with incident diabetes complications were estimated from claims of the largest German statutory health insurance company using panel regression methods that are less likely to yield biased estimates than models based on cross-sectional data [31].

### Results in the context of diabetes care in Germany

As the aim of the analyses was to highlight the long-term health benefits and cost saving potential of improved risk factor control, we did not consider any resources and costs needed to achieve these improvements. However, all real-world risk factor improvement strategies of course come with costs to achieve these improvements. We show that the maximum per-person annual costs for interventions that improve HbA_1c_ control be cost-effective at a willingness-to-pay threshold of €50,000 per QALY gained over a 30-year time horizon range between €190 and €549. In context, the per-person annual costs for metformin, sulfonylurea, sodium–glucose cotransporter 2 (SGLT-2) inhibitors, dipeptidyl peptidase 4 (DPP-4) inhibitors and glucagon-like peptide-1 (GLP-1) antagonists in Germany are approximately €33–99, €31–131, €377–884, €350–511 and €861–1463, respectively [42]. It is important to mention that German guidelines for Health Technology Assessment (HTA) do not endorse the use of cost-effectiveness analyses for reimbursement decisions, and no formal willingness-to-pay threshold for a QALY gained is defined for Germany [43].

### Strengths of this study

We used one of the most established type 2 diabetes simulation models [29]. We parameterised the model using the characteristics of a concurrent population-based diabetes cohort and healthcare expenditures and quality of life variables from large real-world and population-based studies from Germany. We further analysed several ABC distribution shift and guideline-based control scenarios, and provide sensitivity analyses to show the robustness of our results.

### Limitations of this study

First, the results of this modelling study are potentially limited by the underlying model assumptions. We demonstrated in a previous study that the risk equations used in the UKPDS-OM2 predict cardiovascular events well but over-estimate mortality. However, the performance of the model for the prediction of other outcomes remains unknown [29]. Second, we assumed and simulated sustained risk factor improvements over the lifetime of simulated individuals. This may represent an over-simplification as such sustained improvements may be unrealistic for some individuals in the population due to their specific genetic risk profiles and during critical life stages, e.g. menopausal transition, which is associated with higher LDL-cholesterol [44]. Third, the utility decrements used were taken from a large German cross-sectional study. It has been shown that estimates from cross-sectional studies tend to overestimate true causal effects [31]. Fourth, the characteristics of our population-based diabetes sample from a regional cohort study in southern Germany may not be entirely representative for all patients with type 2 diabetes in Germany. As previous studies indicated that control of diabetes risk factors in patients in southern Germany is better than in the rest of Germany [45], the results may underestimate the true health and economic benefits. In addition, this sample only included 146 patients with type 2 diabetes. Fifth, the mean population levels of HbA_1c_, SBP and LDL-cholesterol may have further decreased since 2014. If this is the case, we may have slightly overestimated the health benefits, as the given changes in risk factors in people with lower baseline disease risk would mostly translate into lower absolute health benefits. Sixth, a population-wide shift in HbA_1c_, SBP or LDL-cholesterol may lead to adverse complications such as hypoglycaemia for individuals at the lower end of the HbA_1c_ distribution, but we did not model these potential adverse outcomes. Due to the low proportion of people in these very low ranges, we assume that this would affect our results only marginally. Finally, cost estimates for complications of diabetes and estimates on diabetes prevalence were taken from samples of Germans insured in the statutory health insurance scheme in which approximately 89% of Germans are included. Compared with the statutory health insurance scheme, the per-person cost estimates for treating complications are likely to be higher and the prevalence of type 2 diabetes is likely to be lower in individuals with private health insurance, and thus the estimates for the national healthcare system perspective may be biased in either direction. We again assume that that this would affect our results only marginally.

### Conclusion

Both population-level shifts in risk factor distribution and the achievement of guideline care recommendations for patients exceeding recommended risk factor levels can result in substantial health benefits and cost savings. The expected long-term health and economic benefits described in this study may help German decision makers to assess the clinical effects of interventions and their costs from an efficiency perspective. Using an internationally accepted willingness-to-pay threshold, treatment aiming for better adherence to the guideline-recommended HbA_1c_ level, SBP level and LDL-cholesterol level should not cost more than €549, €760 and €958 a year, respectively.

## Supplementary Information

Below is the link to the electronic supplementary material.

Supplementary file1 (PDF 591 KB)

## Data Availability

The data are subject to national data protection laws, and restrictions were imposed by the Ethics Committee of the Bavarian Medical Association to ensure data privacy of the study participants; therefore, data cannot be made freely available in a public repository. Data are third-party and belong to the KORA research platform, but may be accessed for specific research projects through individual project agreements. Interested researchers can request data from KORA via the online tool (https://www.helmholtz-munich.de/en/epi/cohort/kora). Interested researchers, who agree to the general terms and conditions of the KORA data user agreement, can therefore access the data of KORA in the same way that we did.

## Abbreviations

ABC: HbA_1c_, systolic Blood pressure and LDL-Cholesterol
KORA: Kooperative Gesundheitsforschung in der Region Augsburg/Cooperative Health Research in the Region of Augsburg
QALY: Quality-adjusted life year
SBP: Systolic blood pressure
UKPDS-OM2: UK Prospective Diabetes Study Outcomes Model 2
