# Supplementary material for: Health and economic impact of improved glucose, blood pressure and lipid control among German adults with type 2 diabetes: a modelling study
Source: Diabetologia. 2023 Jun 30;66(9):1693–704. doi: 10.1007/s00125-023-05950-3 (PMC10390361; doi:10.1007/s00125-023-05950-3)
Supplement: Supplementary file 1 — Supplementary file1 (PDF 591 KB) [file 125_2023_5950_MOESM1_ESM.pdf]

Electronic Supplementary Material

Table of Contents

ESM Appendix A. Generation of synthetic population with R package 'Synthpop' 2

ESM Appendix B. Model parameters for health care expenditures 2

ESM Table 1. Age and sex specific annual health care costs in the year of event and follow-up years of the eight complications 2

ESM Appendix C. Model parameters for utility decrements 4

ESM Table 2. Initial utility and utility decrements associated with the eight complications....................... 4

ESM Table 3. Health utility decrements associated with demographic characteristics, type 2 diabetes and cardio- and micro-vascular conditions 6

ESM Appendix D. Per-person effects from improved risk factor control stratified by age and sex 7

ESM Table 4. Per-person costs savings, QALY and Life year gains from improved HbA_{1c}, SBP or LDL level over 5 years, stratified by age and sex 8

ESM Table 5. Per-person costs savings, QALY and Life year gains from improved HbA1c, SBP or LDL level over 10 years, stratified by age and sex 9

ESM Table 6. Per-person costs savings, QALY and Life year gains from improved HbA_{1c}, SBP or LDL level over 30 years, stratified by age and sex 10

ESM Appendix E. Number of type 2 diabetes patients by age and sex in German population 11

ESM Table 7. Population size and prevalence of type 2 diabetes in age and sex subgroups 11

ESM Table 8. Number of adults with type 2 diabetes by age and sex 12

ESM Table 9. Prevalence of adults with type 2 diabetes whose HbA_{1c}, SBP or LDL levels exceed guideline recommended thresholds by age and sex 12

ESM Table 10. Per person costs savings, QALY and life year gains of improved HbA_{1c}, SBP or LDL level over 5, 10, 30 years adjusted for age and sex distribution of German T2D patients... 13

ESM Appendix F. Cumulative event rates of eight complications in the reference and intervention groups over simulation periods of 5, 10 and 30 years 14

ESM Table 11. Cumulative event rates of eight complications over 5 years 14

ESM Table 12. Cumulative event rates of eight complications over 10 years 15

ESM Table 13. Cumulative event rates of eight complications over 30 years 16

ESM Table 14. Per person costs savings, QALY gains and life years gains from improved HbA_{1c}, SBP or LDL level over 5, 10, 30 years (with 95%-confidence interval) 18

ESM Appendix G. Tornado plots showing one-way sensitivity analysis by alternative discount rates, model parameters and number of internal loops... 21

References 23

ESM Appendix A. Generation of synthetic population with R package 'Synthpop'

The generation of the synthetic data assumes that the observed data is a sample from a population with parameters that can be estimated by the synthesizer. The synthesizer fits the data to the assumed distribution and obtains estimates of its parameters (1). We compared the correlations between risk factors and demographic characteristics of the new sample with those observed in the original KORA-S4 sample using graphs and correlation coefficients. The distributions of variables and the correlations between them in the synthetic sample were mostly preserved from the original KORA-S4 sample.

ESM Appendix B. Model parameters for health care expenditures

We extracted the annual health care expenditures associated with eight complications: IHD (Ischemic heart disease), MI (Myocardial infarction), Heart failure, Stroke, Amputation, Blindness, Renal failure and Ulcer, for type 2 diabetes patients in Germany stratified by age and sex, at the year of events and in subsequent years, as input parameters for our model from 'Supplementary Table 8' of Kähm et al. 2018 (2).

For 'Heart Failure', we used the age and sex specific health care expenditure estimates of 'CHF (Chronic Heart Failure)' in the paper, as equivalent; For 'Renal Failure', we used the estimates of 'ESRD' (End Stage Renal Disease) as equivalent; For 'Ulcer', we used the estimates of 'Diabetic foot' as equivalent.

The age and sex specific annual health care costs associated with incident complications in patients with type 2 diabetes estimated from German population that we used as model parameters in UKPDS OM2 are presented in ESM Table 1.

ESM Table 1. Age and sex specific annual health care costs in the year of event and follow-up years of the eight complications

Type of Complication	Age group (In years)	Annualized total costs (in €) in the year of event and follow-up years					
		Male			Female		
		Year of event		Follow-up years	Year of event		Follow-up years
		Fatal	Non-fatal		Fatal	Non-fatal	
**IHD**	**<50**	24,209	7287	2080	10,124	8894	7092
	**50-59**	24,319	9401	4649	10,054	11,110	4469
	**60-69**	24,475	9296	4362	10,038	8563	3614
	**70-79**	24,665	9123	3703	10,156	8806	4957
	**>80**	24,466	8225	4186	9741	8195	4832
**MI**	**<50**	10,515	10,386	4765	6554	9302	5569
	**50-59**	10,624	10,105	4289	6483	9895	4509
	**60-69**	10,780	11,184	4621	6468	10,341	6218
	**70-79**	10,970	11,728	5577	6585	12,060	6146
	**>80**	10,771	9203	4130	6171	8157	3626
**Heart failure**	**<50**	-	6775	5718	-	7527	6896
	**50-59**	-	6882	5598	-	6891	5570
	**60-69**	-	6874	5696	-	7237	6197
	**70-79**	-	7551	5977	-	7474	5923
	**>80**	-	6343	5139	-	5751	4101
**Stroke**	**<50**	12,765	13,584	7958	11,263	24,804	34,123
	**50-59**	12,874	13,459	7478	11,193	15,575	14,036
	**60-69**	13,031	14,684	10,070	11,177	15,018	10,534
	**70-79**	13,220	14,391	9361	11,295	14,781	9804
	**>80**	13,021	12,465	6014	10,880	11,592	5979
**Amputation**	**<50**	-	19,368	11,594	-	19,823	12,994
	**50-59**	-	19,562	11,788	-	19,710	12,882
	**60-69**	-	19,841	12,066	-	19,686	12,857
	**70-79**	-	20,178	12,403	-	19,873	13,045
	**>80**	-	19,824	12,049	-	19,210	12,381
**Blindness**	**<50**	-	4669	3483	-	6361	5097
	**50-59**	-	4864	3677	-	6248	4985
	**60-69**	-	5142	3955	-	6224	4960
	**70-79**	-	5479	4292	-	6411	5148
	**>80**	-	5125	3939	-	5748	4484
**Renal failure**	**<50**	-	34,075	24,189	-	26,614	19,706
	**50-59**	-	34,269	24,384	-	26,502	19,594
	**60-69**	-	34,547	24,662	-	26,478	19,569
	**70-79**	-	34,884	24,999	-	26,665	19,756
	**>80**	-	34,530	24,645	-	26,002	19,093
**Ulcer**	**<50**	-	3314	3313	-	4743	5309
	**50-59**	-	3429	3862	-	3873	4240
	**60-69**	-	3629	4034	-	3972	4396
	**70-79**	-	4228	4393	-	4109	4350
	**>80**	-	3838	3649	-	3419	3057
**No complication**	**<50**	-	2102		-	3001	
	**50-59**	-	2296		-	2889	
	**60-69**	-	2574		-	2864	
	**70-79**	-	2911		-	3052	
	**>80**	-	2558		-	2388	

ESM Appendix C. Model parameters for utility decrements

We extracted utility decrements associated with IHD (Ischemic heart disease), MI (Myocardial infarction), Heart Failure, Stroke, Blindness, and Ulcer for type 2 diabetes patients in Germany as input parameters for our model from the results of Laxy et al. 2021 (3). As utility decrements for amputation and renal failure were not reported in this source, and we were also unable to identify the respective values for a German population through other sources, we kept the default parameters of utility decrements of complications in UKPDS Outcome Model 2. The estimated utility decrement for amputation was derived from UKPDS patients and published in 2014 (4); the estimate for renal failure was from a meta-analysis of quality of life studies (5). The utility decrements associated diabetes complications estimated from German populations that we used as model parameters in UKPDS OM2 are presented in ESM Table 2.

ESM Table 2. Initial utility and utility decrements associated with the eight complications

	Utility decrements		Source
	At time of event	In subsequent years	
**IHD :**	0.000	0.000	Laxy et al, Value in Health 2020
**MI :**	0.000	0.000	Laxy et al, Value in Health 2020
**Heart failure :**	-0.107	-0.107	Laxy et al, Value in Health 2020
**Stroke :**	-0.122	-0.122	Laxy et al, Value in Health 2020
**Amputation :**	-0.172	-0.172	Default UKPDS
**Blindness :**	-0.094	-0.094	Laxy et al, Value in Health 2020
**Renal failure :**	-0.330	-0.330	Default UKPDS
**Ulcer :**	-0.042	-0.042	Laxy et al, Value in Health 2020
**Initial utility :**	0.815		Laxy et al, Value in Health 2020

About the estimated initial utility in German population: Laxy et al. 2021 reported the health utility decrements associated with type 2 diabetes and cardio- and micro-vascular conditions, estimated from a multivariate linear regression model with an interaction term between diabetes

and cardiovascular or microvascular complications (ESM Table 3). In addition, the utility decrements (coefficients) associated with the demographic factors (age, sex, etc.) and comorbidities (cancer, asthma, etc.) can be extracted. From the sample characteristics (proportions of binary/categorical variables and means of continuous variables) and the regression coefficients we derived the utility value for an average person with type 2 diabetes, which we assumed to be representative for the utility in of an adult with Type 2 diabetes in Germany.

ESM Table 3. Health utility decrements associated with demographic characteristics, type 2 diabetes and cardio- and micro-vascular conditions

	Health utility decrement associated with type 2 diabetes and cardio- and micro-vascular conditions (Table 2. Laxy et al. 2021)			Characteristics of KORA-S4 sample
	ß	SE	p-value	Proportion or mean
Intercept	1.187	0.019	<.0001	
Telephone Interview	-0.044	0.007	<.0001	13.7%
Age (years)	-0.003	0.000	<.0001	62.1
Female	-0.029	0.004	<.0001	46%
Education				
Medium	0.009	0.004	0.0483	25.8%
High	0.034	0.006	<.0001	10.4%
BMI	-0.003	0.000	<.0001	31.9
Smoking	-0.017	0.006	0.0042	14%
Cancer	-0.025	0.006	<.0001	14.7%
Asthma	-0.047	0.008	<.0001	9.1%
Chronic bronchitis	-0.032	0.008	<.0001	11.9%
COPD	-0.085	0.012	<.0001	5.3%
Diabetes	-0.028	0.014	0.0357	100%
Hypertension	0.029	0.014	0.037	77.8%
MI	0.002	0.020	0.9038	16%
Cardiac arrhythmia	-0.030	0.015	0.0412	22.6%
HF	-0.107	0.019	<.0001	14%
CHD	0.002	0.018	0.9055	9%
Stroke	-0.122	0.018	<.0001	6%
Neuropathy	-0.067	0.020	0.0017	21.3%
PVD	-0.025	0.033	0.4467	5.9%
Diabetic foot	-0.042	0.030	0.1663	0%
Nephropathy	-0.032	0.025	0.2189	10.5%
Blindness	-0.094	0.056	0.091	1%

ESM Appendix D. Per-person effects from improved risk factor control stratified by age and sex

To explore the health outcomes in age and sex subgroups, we stratified the study population by age (<60 years / 60-70 years / >70 years) and sex (male / female) and calculated simulation effects in each subgroup. The primary reason for this age categorization was to ensure a sufficient sample size in each subgroup, in order to derive stable results from the simulation. Per-person cost savings, QALY and life years gains by age and sex from improved ABC level over 5, 10, and 30 years are presented in ESM Table 4-6.

ESM Table 4. Per-person costs savings, QALY and Life year gains from improved HbA_{1c}, SBP or LDL level over 5 years, stratified by age and sex

	< 60 years						60 -70 years						> 70 years					
	F	M	F	M	F	M	F	M	F	M	F	M	F	M	F	M	F	M
	Total costs		Total QALY		Total LYs		Total costs		Total QALY		Total LYs		Total costs		Total QALY		Total LYs	
Reference Group[a]	36,252	31,694	3.42	3.37	4.34	4.30	29,718	28,052	3.35	3.26	4.20	4.09	31,289	31,773	2.91	2.79	3.64	3.51
	Δ Cost		Δ QALY		Δ Lys		Δ Cost		Δ QALY		Δ Lys		Δ Cost		Δ QALY		Δ Lys	
HbA_{1c} -5.5 mmol/mol (-0.5%)	6	67	0.002	0.002	0.002	0.001	39	105	0.003	0.004	0.003	0.004	85	113	0.003	0.004	0.002	0.004
HbA_{1c} GLC	5	361	0.006	0.006	0.007	0.004	130	270	0.010	0.010	0.010	0.010	245	419	0.005	0.015	0.002	0.015
SBP -10mmHg	45	131	0.002	0.003	0.003	0.003	82	199	0.005	0.007	0.005	0.007	111	204	0.006	0.006	0.006	0.005
SBP GLC	-134	443	0.009	0.003	0.014	-0.001	279	398	0.010	0.010	0.009	0.010	396	415	0.008	0.005	0.006	0.003
LDL -0.26mmol/l	-10	94	0.002	0.002	0.003	0.002	38	109	0.003	0.006	0.004	0.007	63	74	0.003	0.006	0.003	0.007
LDL GLC	83	304	0.009	0.014	0.011	0.016	137	486	0.015	0.027	0.017	0.032	248	515	0.009	0.027	0.009	0.031
Combined GLC	137	381	0.009	0.014	0.010	0.016	256	640	0.017	0.027	0.018	0.030	265	552	0.015	0.030	0.016	0.033
Combined Reduction	95	259	0.006	0.007	0.006	0.007	159	373	0.011	0.016	0.011	0.017	247	363	0.011	0.018	0.011	0.020

[a] Reference Group: group without improvement in HbA_{1c}, SBP and LDL

Abbreviations: F: Female, M: Male; Cost: costs of complications in the reference group; QALY: quality-adjusted life years in the reference group; Δ C: cumulative cost savings; Δ Q: cumulative QALYs gained; GLC: Guideline Care Intervention

ESM Table 5. Per-person costs savings, QALY and Life year gains from improved HbA1c, SBP or LDL level over 10 years, stratified by age and sex

	< 60 years						60 -70 years						> 70 years					
	F	M	F	M	F	M	F	M	F	M	F	M	F	M	F	M	F	M
	Total costs		Total QALY		Total LYs		Total costs		Total QALY		Total LYs		Total costs		Total QALY		Total LYs	
Reference Group[a]	63,005	55,999	6.0	5.9	7.7	7.6	50,451	47,588	5.7	5.5	7.2	6.9	47,404	47,469	4.7	4.5	5.9	5.6
	Δ Cost		Δ QALY		Δ Lys		Δ Cost		Δ QALY		Δ Lys		Δ Cost		Δ QALY		Δ Lys	
HbA_{1c} -5.5 mmol/mol (-0.5%)	92	86	0.01	0.01	0.00	0.01	84	194	0.01	0.01	0.01	0.01	100	131	0.01	0.02	0.01	0.02
HbA_{1c} GLC	319	541	0.02	0.03	0.02	0.03	380	483	0.03	0.04	0.03	0.04	237	912	0.02	0.03	0.02	0.03
SBP -10mmHg	197	192	0.01	0.01	0.01	0.01	134	348	0.02	0.03	0.02	0.03	74	407	0.03	0.02	0.03	0.02
SBP GLC	-326	316	0.04	0.03	0.04	0.03	265	729	0.05	0.05	0.06	0.06	232	675	0.10	0.03	0.11	0.03
LDL -0.26mmol/l	28	18	0.01	0.01	0.01	0.02	-30	101	0.01	0.02	0.02	0.03	-112	130	0.02	0.02	0.03	0.02
LDL GLC	50	300	0.04	0.05	0.04	0.06	153	594	0.05	0.09	0.06	0.12	169	517	0.05	0.09	0.05	0.11
Combined GLC	323	551	0.03	0.05	0.04	0.06	416	913	0.06	0.10	0.07	0.12	246	726	0.06	0.10	0.07	0.11
Combined Reduction	252	405	0.02	0.03	0.02	0.03	293	636	0.04	0.06	0.04	0.06	250	502	0.05	0.06	0.05	0.07

[a] Reference Group: group without improvement in HbA_{1c}, SBP and LDL

Abbreviations: F: Female, M: Male; Cost: costs of complications in the reference group; QALY: quality-adjusted life years in the reference group; Δ C: cumulative cost savings; Δ Q: cumulative QALYs gained; GLC: Guideline Care Intervention

ESM Table 6. Per-person costs savings, QALY and Life year gains from improved HbA_{1c}, SBP or LDL level over 30 years, stratified by age and sex

	< 60 years						60 -70 years						> 70 years					
	F	M	F	M	F	M	F	M	F	M	F	M	F	M	F	M	F	M
	Total costs		Total QALY		Total LYs		Total costs		Total QALY		Total LYs		Total costs		Total QALY		Total LYs	
Reference Group[a]	111,513	102,741	11.1	10.5	14.1	13.5	74,953	70,724	8.9	8.1	11.3	10.3	60,366	60,638	6.5	6.0	8.2	7.5
	Δ Cost		Δ QALY		Δ Lys		Δ Cost		Δ QALY		Δ Lys		Δ Cost		Δ QALY		Δ Lys	
HbA_{1c} -5.5 mmol/mol (-0.5%)	172	419	0.03	0.03	0.03	0.03	147	199	0.04	0.04	0.04	0.05	138	84	0.02	0.04	0.02	0.05
HbA_{1c} GLC	816	1298	0.09	0.12	0.09	0.12	524	637	0.11	0.13	0.11	0.14	326	445	0.06	0.13	0.07	0.14
SBP -10mmHg	302	362	0.04	0.06	0.05	0.07	150	379	0.07	0.08	0.09	0.10	271	252	0.05	0.07	0.06	0.08
SBP GLC	-1007	2465	0.10	0.08	0.13	0.05	415	804	0.20	0.18	0.23	0.21	819	214	0.10	0.11	0.11	0.13
LDL -0.26mmol/l	-21	42	0.03	0.05	0.04	0.06	-101	-41	0.05	0.07	0.06	0.09	-37	-41	0.03	0.06	0.04	0.07
LDL GLC	-292	177	0.16	0.21	0.20	0.26	-312	6	0.19	0.29	0.24	0.36	-40	-140	0.12	0.25	0.15	0.31
Combined GLC	509	789	0.16	0.23	0.18	0.28	220	708	0.24	0.33	0.28	0.39	200	144	0.16	0.26	0.18	0.31
Combined Reduction	359	738	0.11	0.14	0.13	0.16	300	532	0.15	0.19	0.17	0.22	306	309	0.12	0.16	0.13	0.19

[a] Reference Group: group without improvement in HbA_{1c}, SBP and LDL

Abbreviations: F: Female, M: Male; Cost: costs of complications in the reference group; QALY: quality-adjusted life years in the reference group; Δ C: cumulative cost savings; Δ Q: cumulative QALYs gained; GLC: Guideline Care Intervention

ESM Appendix E. Number of type 2 diabetes patients by age and sex in German population

To estimate the number of adults with type 2 diabetes by age and sex in Germany, population size and prevalence of type 2 diabetes in each age and sex subgroup is required. We extracted population size estimates from a paper that reported adjusted inter-censal German population estimates (data from year 2010 was used) (6), and extracted the prevalence of type 2 diabetes from a study that used ICD-coded diagnosis from statutory health insurance data in Germany from year 2010 (7). Details are presented in ESM Table 7.

With the population statistics available in finer groups, we were able to calculate the number of people with type 2 diabetes in the pre-specified subgroups, shown in ESM Table 8.

ESM Table 7. Population size and prevalence of type 2 diabetes in age and sex subgroups

Age	Sex	Prevalence of type 2 diabetes (%)	Population size
20-39 years	Female	1.3[a]	9,530,438
	Male	1.6[a]	9,703,400
40–49 years	Female	1.3	6,630,707
	Male	1.6	6,789,150
50–59 years	Female	3.6	5,790,026
	Male	5.7	5,737,229
60–69 years	Female	10.0	4,598,049
	Male	14.5	4,319,805
70–79 years	Female	16.9	4,446,196
	Male	21.9	3,626,000
80–89 years	Female	24.0	2,424,930
	Male	26.3	1,230,095
90–99 years	Female	24.9	393,950
	Male	24.1	98,239

[a] In the paper 'The prevalence and incidence of diabetes in Germany'(7), the prevalence of type 2 diabetes was only reported for age groups over 40 years of age. We assumed the lowest prevalence reported as the prevalence for the younger age group (20-39 years).

ESM Table 8. Number of adults with type 2 diabetes by age and sex

Age	Sex	Summed population size	Pooled prevalence of type 2 diabetes (%)	Number of adults with type 2 diabetes	Proportion of adults with type 2 diabetes in each group (%)
<60	Female	21,951,171	1.9	418,536	9.0
	Male	22,229,779	2.7	590,903	12.7
60-70	Female	4,598,049	10.0	459,805	9.8
	Male	4,319,805	14.5	626,372	13.4
>70	Female	7,265,076	19.7	1,431,484	30.7
	Male	4,954,334	23.0	1,141,285	24.4
Total		65,318,214	7.1	4,668,384	100.0

ESM Table 9. Prevalence of adults with type 2 diabetes whose HbA_{1c}, SBP or LDL levels exceed guideline recommended thresholds by age and sex

Age	Sex	Prevalence of HbA_{1c} > 53 mmol/mol (7%) [%]	Prevalence of SBP > 140 mmHg (%)	Prevalence of LDL > 2.60 mmol/l (%)	Prevalence of either HbA_{1c}, SBP or LDL exceeds threshold (%)
<60	Female	34	8	49	70
	Male	26	9	52	66
60-70	Female	45	22	67	86
	Male	33	31	64	84
>70	Female	40	12	72	84
	Male	23	32	61	79
Overall		34	20	61	79

ESM Table 10. Per person costs savings, QALY and life year gains of improved HbA_{1c}, SBP or LDL level over 5, 10, 30 years adjusted for age and sex distribution of German T2D patients

	5 YEARS			10 YEARS			30 YEARS		
	Cost	QALY	LY	Cost	QALY	LY	Cost	QALY	LY
Reference Group[a]	31,326	3.1	3.9	50,270	5.1	6.5	73,349	7.8	9.8
HbA_{1c} [mmol/mol]	Δ Cost	Δ QALY	Δ LYs	Δ Cost	Δ QALY	Δ LYs	Δ Cost	Δ QALY	Δ LYs
-5.5 (-0.5%)	80	0.003	0.003	100	0.01	0.01	173	0.03	0.04
Guideline Care: ≤53 (≤7%)	272	0.009	0.008	446	0.03	0.03	585	0.10	0.11
Systolic Blood Pressure [mmHG]	Δ Cost	Δ QALY	Δ LYs	Δ Cost	Δ QALY	Δ LYs	Δ Cost	Δ QALY	Δ LYs
-10	139	0.005	0.005	207	0.02	0.02	283	0.06	0.07
Guideline Care: ≤140	348	0.007	0.006	317	0.06	0.06	681	0.12	0.13
LDL Cholesterol [mmol/L]	Δ Cost	Δ QALY	Δ LYs	Δ Cost	Δ QALY	Δ LYs	Δ Cost	Δ QALY	Δ LYs
-0.26	67	0.004	0.004	-4	0.02	0.02	-33	0.05	0.06
Guideline Care: ≤2.60	324	0.017	0.019	288	0.06	0.08	-80	0.20	0.24
Combined Guideline Care	385	0.020	0.022	493	0.07	0.08	359	0.22	0.26
COMBINED POPULATION-WIDE REDUCTION	270	0.012	0.013	370	0.04	0.05	396	0.14	0.16

[a] Reference Group: group without improvement in HbA_{1c}, SBP and LDL
Abbreviations: Cost: costs of complications in the reference group; QALY: quality-adjusted life years in the reference group; LY: life years in the reference group; Δ Cost: cumulative cost savings; Δ QALY: cumulative QALY gained; Δ LYs: Life-years gained

ESM Appendix F. Cumulative event rates of eight complications in the reference and intervention groups over simulation periods of 5, 10 and 30 years

ESM Table 11. Cumulative event rates of eight complications over 5 years

		IHD	MI	HF	STK	AMP	BLN	RF	ULC
Cumulative event rates in the Reference Group		0.1260	0.0546	0.1777	0.0249	0.0160	0.0136	0.0256	0.0071
Intervention Groups		Δ	Δ	Δ	Δ	Δ	Δ	Δ	Δ
HbA_{1c}	-3.3 (-0.3%)	0.0001	-0.0011	0.0000	-0.0005	-0.0008	-0.0007	0.0000	-0.0003
mmol/mol	-5.5 (-0.5%)	0.0000	-0.0017	0.0000	-0.0008	-0.0012	-0.0011	0.0000	-0.0005
	-11 (-1.0%)	0.0000	-0.0036	-0.0001	-0.0017	-0.0023	-0.0021	0.0000	-0.0010
Guideline Care:	≤53 (≤7%)	0.0000	-0.0017	-0.0001	-0.0007	-0.0010	-0.0012	0.0000	-0.0005
SBP	-5	-0.0009	-0.0010	0.0001	-0.0016	-0.0002	-0.0004	0.0000	0.0000
mmHg	-10	-0.0018	-0.0018	0.0000	-0.0031	-0.0004	-0.0008	-0.0001	0.0000
	-15	-0.0026	-0.0026	0.0000	-0.0045	-0.0006	-0.0012	-0.0001	0.0000
Guideline Care:	≤140	-0.0007	-0.0007	0.0000	-0.0017	-0.0002	-0.0004	0.0000	0.0000
LDL	-0.13	-0.0009	-0.0013	-0.0003	-0.0004	0.0000	0.0001	0.0000	0.0000
mmol/l	-0.26	-0.0019	-0.0027	-0.0005	-0.0007	0.0000	0.0000	0.0000	0.0000
	-0.39	-0.0028	-0.0040	-0.0008	-0.0011	0.0000	0.0000	-0.0001	0.0000

Guideline Care: ≤2.60	-0.0053	-0.0077	-0.0013	-0.0021	0.0000	0.0000	-0.0002	0.0001
Combined Guideline Care	-0.0058	-0.0099	-0.0013	-0.0042	-0.0011	-0.0015	-0.0002	-0.0005
**COMBINED POPULATION-WIDE REDUCTION**	**-0.0035**	**-0.0059**	**-0.0005**	**-0.0045**	**-0.0015**	**-0.0018**	**-0.0001**	**-0.0005**

Δ: difference in cumulative event rates [intervention group vs. reference group]
Abbreviations: IHD (ischaemic Heart Disease), MI (myocardial infarction), HF (heart failure), STK (stroke), AMP (amputation), BLN (blindness), RF (renal failure), ULC (ulcer)

ESM Table 12. Cumulative event rates of eight complications over 10 years

		**IHD**	**MI**	**HF**	**STK**	**AMP**	**BLN**	**RF**	**ULC**
Cumulative event rates Reference Group		0.1560	0.1010	0.1959	0.0502	0.0286	0.0241	0.0262	0.0128
Intervention Groups		Δ	Δ	Δ	Δ	Δ	Δ	Δ	Δ
HbA_{1c}	-3.3 (-0.3%)	0.0001	-0.0022	0.0001	-0.0010	-0.0013	-0.0012	0.0000	-0.0006
mmol/mol	-5.5 (-0.5%)	0.0002	-0.0036	0.0000	-0.0016	-0.0021	-0.0018	0.0000	-0.0010
	-11 (-1.0%)	0.0000	-0.0068	0.0000	-0.0033	-0.0042	-0.0036	0.0000	-0.0018
Guideline Care: ≤53 (≤7%)		0.0000	-0.0035	0.0000	-0.0014	-0.0022	-0.0020	0.0000	-0.0009
SBP	-5	-0.0017	-0.0017	0.0000	-0.0031	-0.0005	-0.0008	-0.0001	0.0000
mmHg	-10	-0.0034	-0.0036	0.0002	-0.0062	-0.0009	-0.0015	-0.0001	0.0001
	-15	-0.0047	-0.0052	0.0003	-0.0089	-0.0012	-0.0022	-0.0002	0.0001

Guideline Care: ≤140		-0.0013	-0.0015	0.0001	-0.0035	-0.0004	-0.0007	-0.0001	0.0000
LDL	-0.13	-0.0018	-0.0027	-0.0004	-0.0007	0.0000	0.0000	-0.0001	0.0001
mmol/l	-0.26	-0.0034	-0.0050	-0.0009	-0.0014	0.0001	0.0001	-0.0001	0.0001
	-0.39	-0.0051	-0.0072	-0.0014	-0.0022	0.0003	0.0001	-0.0001	0.0002
Guideline Care: ≤2.60		-0.0094	-0.0137	-0.0024	-0.0039	0.0002	0.0001	-0.0003	0.0002
Guideline Care ABC		-0.0106	-0.0179	-0.0022	-0.0081	-0.0023	-0.0025	-0.0003	-0.0006
**COMBINED POPULATION-WIDE REDUCTION**		**-0.0064**	**-0.0115**	**-0.0008**	**-0.0088**	**-0.0029**	**-0.0033**	**-0.0002**	**-0.0009**

Δ: difference in cumulative event rates [intervention group vs. reference group]
Abbreviations: IHD (ischaemic Heart Disease), MI (myocardial infarction), HF (heart failure), STK (stroke), AMP (amputation), BLN (blindness), RF (renal failure), ULC (ulcer)

ESM Table 13. Cumulative event rates of eight complications over 30 years

	IHD	MI	HF	STK	AMP	BLN	RF	ULC
Cumulative event rates Reference Group	0.2169	0.1931	0.2379	0.1064	0.0557	0.0428	0.0270	0.0231
Intervention Groups	Δ	Δ	Δ	Δ	Δ	Δ	Δ	Δ

HbA_{1c}	-3.3 (-0.3%)	0.0006	-0.0033	0.0001	-0.0020	-0.0029	-0.0018	0.0000	-0.0010
mmol/mol	-5.5 (-0.5%)	0.0007	-0.0057	0.0002	-0.0031	-0.0046	-0.0031	0.0000	-0.0016
	-11 (-1.0%)	0.0010	-0.0116	0.0004	-0.0064	-0.0088	-0.0061	0.0000	-0.0031
Guideline Care:	≤53 (≤7%)	0.0007	-0.0056	0.0001	-0.0027	-0.0053	-0.0033	0.0000	-0.0016
SBP	-5	-0.0024	-0.0031	0.0004	-0.0064	-0.0012	-0.0012	-0.0001	0.0002
mmHg	-10	-0.0052	-0.0056	0.0008	-0.0124	-0.0022	-0.0023	-0.0001	0.0002
	-15	-0.0078	-0.0086	0.0013	-0.0179	-0.0030	-0.0034	-0.0002	0.0004
Guideline Care:	≤140	-0.0017	-0.0018	0.0003	-0.0066	-0.0009	-0.0007	-0.0001	0.0001
LDL	-0.13	-0.0029	-0.0040	-0.0010	-0.0015	0.0002	0.0003	-0.0001	0.0001
mmol/l	-0.26	-0.0059	-0.0079	-0.0018	-0.0029	0.0005	0.0002	-0.0001	0.0002
	-0.39	-0.0087	-0.0118	-0.0024	-0.0037	0.0007	0.0004	-0.0002	0.0003
Guideline Care:	≤2.60	-0.0150	-0.0215	-0.0037	-0.0065	0.0012	0.0008	-0.0004	0.0006
Guideline Care ABC		-0.0163	-0.0288	-0.0030	-0.0149	-0.0050	-0.0037	-0.0004	-0.0009
**COMBINED POPULATION-WIDE REDUCTION**		**-0.0104**	**-0.0192**	**-0.0005**	**-0.0173**	**-0.0058**	**-0.0051**	**-0.0003**	**-0.0013**

Δ: difference in cumulative event rates [intervention group vs. reference group]

Abbreviations: IHD (ischaemic Heart Disease), MI (myocardial infarction), HF (heart failure), STK (stroke), AMP (amputation), BLN (blindness), RF (renal failure), ULC (ulcer)

ESM Table 14. Per person costs savings, QALY gains and life years gains from improved HbA_{1c}, SBP or LDL level over 5, 10, 30 years (with 95%-confidence interval)

	5 YEARS			10 YEARS			30 YEARS		
	Cost	QALY	LY	Cost	QALY	LY	Cost	QALY	LY
Reference Group[a]	30,987	3.2	4.1	51,821	5.5	6.9	80,528	8.7	11.0
**HbA_{1c} [mmol/mol]**	**Δ Cost**	**Δ QALY**	**Δ LYs**	**Δ Cost**	**Δ QALY**	**Δ LYs**	**Δ Cost**	**Δ QALY**	**Δ LYs**
-3.3 (-0.3%)	45 (21, 63)	0.002 (0.001, 0.003)	0.002 (0.001, 0.003)	52 (41, 126)	0.007 (0.004, 0.008)	0.007 (0.004, 0.009)	96 (40, 195)	0.023 (0.018, 0.03)	0.02 (0.02, 0.03)
-5.5 (-0.5%)	69 (43, 92)	0.003 (0.002, 0.004)	0.003 (0.002, 0.004)	121 (83, 182)	0.01 (0.007, 0.013)	0.01 (0.07, 0.013)	195 (80, 293)	0.04 (0.03, 0.05)	0.04 (0.03, 0.05)
-11 (-1.0%)	139 (94, 174)	0.006 (0.004, 0.007)	0.005 (0.004, 0.007)	265 (175, 339)	0.02 (0.016, 0.025)	0.02 (0.015, 0.025)	411 (182, 554)	0.07 (0.06, 0.09)	0.08 (0.06, 0.1)
Guideline Care: ≤53 (≤7%)	211 (154, 297)	0.009 (0.005, 0.011)	0.009 (0.004, 0.012)	451 (344, 676)	0.03 (0.02, 0.04)	0.03 (0.02, 0.04)	664 (518, 1138)	0.11 (0.08, 0.14)	0.12 (0.09, 0.15)
**Systolic Blood Pressure [mmHG]**	**Δ Cost**	**Δ QALY**	**Δ LYs**	**Δ Cost**	**Δ QALY**	**Δ LYs**	**Δ Cost**	**Δ QALY**	**Δ LYs**

-5	71 (44, 94)	0.003 (0.002, 0.004)	0.003 (0.002, 0.004)	116 (62, 179)	0.01 (0.007, 0.012)	0.01 (0.007, 0.014)	134 (51, 232)	0.04 (0.03, 0.05)	0.04 (0.03, 0.05)
-10	132 (90, 167)	0.005 (0.004, 0.007)	0.005 (0.003, 0.007)	238 (154, 307)	0.02 (0.014, 0.023)	0.02 (0.01, 0.03)	288 (128, 408)	0.07 (0.05, 0.09)	0.08 (0.06, 0.1)
-15	194 (143, 238)	0.007 (0.005, 0.01)	0.007 (0.005, 0.01)	360 (233, 444)	0.03 (0.02, 0.03)	0.03 (0.02, 0.04)	424 (174, 580)	0.10 (0.08, 0.13)	0.11 (0.09, 0.15)
Guideline Care: ≤ 140	353 (234, 470)	0.009 (0.008, 0.018)	0.008 (0.007, 0.019)	507 (349, 922)	0.05 (0.03, 0.06)	0.05 (0.03, 0.07)	605 (179, 1154)	0.16 (0.12, 0.20)	0.18 (0.13, 0.23)

**LDL Cholesterol [mmol/L]**	**Δ Cost**	**Δ QALY**	**Δ LYs**	**Δ Cost**	**Δ QALY**	**Δ LYs**	**Δ Cost**	**Δ QALY**	**Δ LYs**
-0.13	27 (13, 54)	0.002 (0.001, 0.003)	0.002 (0.001, 0.004)	1 (-9,70)	0.007 (0.005, 0.01)	0.01 (0.006, 0.012)	-58 (-115, 25)	0.03 (0.02, 0.03)	0.03 (0.02, 0.04)
-0.26	64 (29,90)	0.004 (0.003, 0.005)	0.005 (0.003, 0.006)	34 (29, 115)	0.015 (0.011, 0.018)	0.018 (0.013, 0.022)	-39 (-115, 29)	0.05 (0.04, 0.06)	0.06 (0.05, 0.08)
-0.39	87 (54, 119)	0.006 (0.005, 0.008)	0.007 (0.006, 0.01)	66 (59, 175)	0.02 (0.02, 0.03)	0.026 (0.02, 0.03)	-71 (-142, 33)	0.07 (0.06, 0.09)	0.09 (0.07, 0.11)

Guideine Care: ≤ 2.60	306 (203, 396)	0.018 (0.015, 0.024)	0.021 (0.017, 0.028)	327 (230, 515)	0.06 (0.05, 0.08)	0.08 (0.06, 0.10)	-111 (-352, 141)	0.22 (0.18, 0.28)	0.27 (0.22, 0.35)
COMBINED GUIDELINE CARE	400 (316, 476)	0.020 (0.018, 0.024)	0.022 (0.019, 0.027)	581 (462, 776)	0.07 (0.06, 0.08)	0.08 (0.07, 0.10)	451 (170, 695)	0.25 (0.22, 0.29)	0.29 (0.26, 0.35)
COMBINED POPULATION-WIDE REDUCTION	254 (202, 294)	0.012 (0.010, 0.014)	0.012 (0.011, 0.015)	422 (339, 514)	0.042 (0.036, 0.047)	0.046 (0.040, 0.052)	438 (190, 589)	0.15 (0.135, 0.172)	0.17 (0.156, 0.201)

[a] Reference Group: group without improvement in HbA_{1c}, SBP and LDL
Abbreviations: Cost: costs of complications in the reference group; QALY: quality-adjusted life years in the reference group; LY: life years in the reference group; Δ Cost: cumulative cost savings; Δ QALY: cumulative QALY gained; Δ LYs: Life-years gained.

ESM Appendix G. Tornado plots showing one-way sensitivity analysis by alternative discount rates, model parameters and number of internal loops

Tornado plots below show one-way sensitivity analysis of change in long-term cost savings (a) and QALY gains (b) from improved ABC, arising from alternative discount rates being 0% and 5% (vs. 3.5%), alternative model parameters of complications health care expenditures and utility decrements being double and half of the current values, and alternative number of internal loops being 5,000 and 20,000 (vs. 10,000).

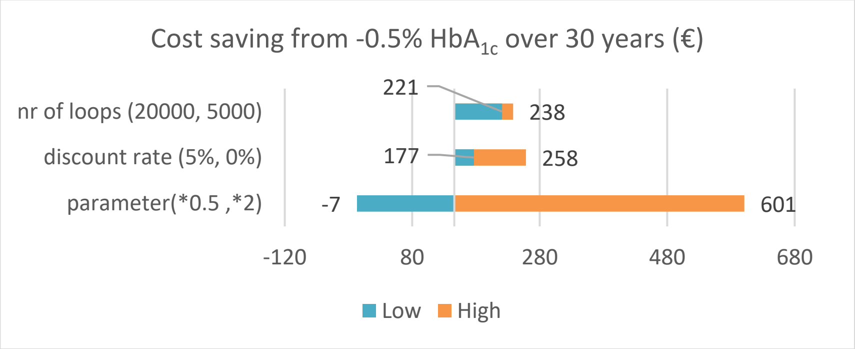

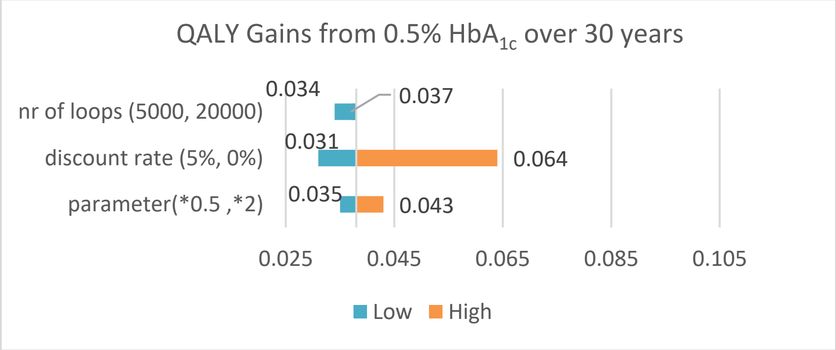

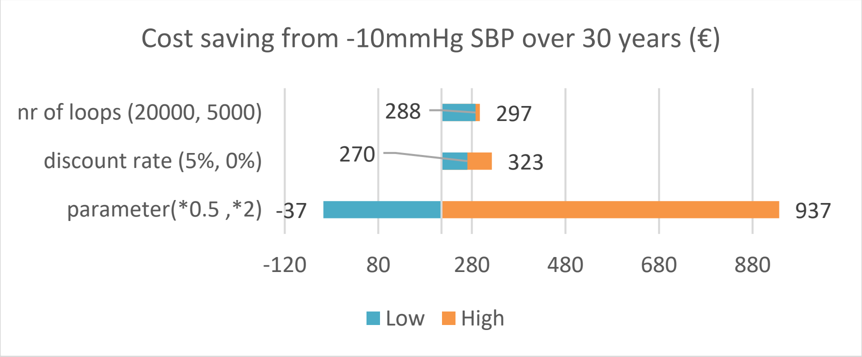

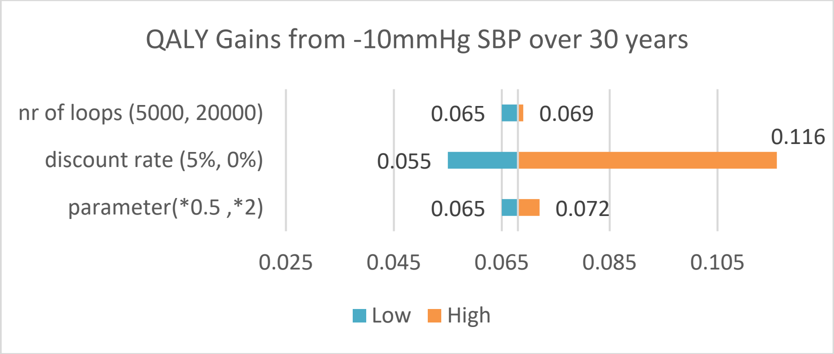

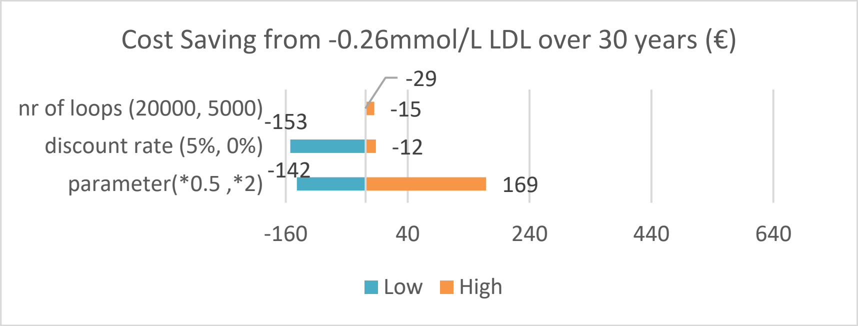

ESM Fig. 1 a) One way sensitivity analysis of changes in cost savings

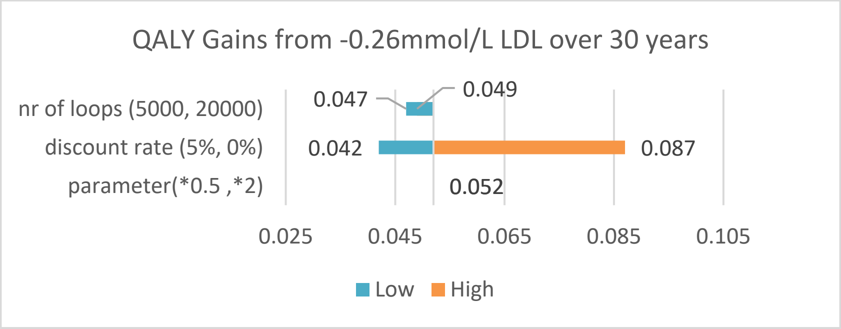

ESM Fig. 1 b) One way sensitivity analysis of changes in QALYs gained

References

- 1. Nowok B, Raab GM, Dibben C. synthpop: Bespoke creation of synthetic data in R. Journal of statistical software. 2016;74:1-26.
- 2. Kahm K, Laxy M, Schneider U, Rogowski WH, Lhachimi SK, Holle R. Health Care Costs Associated With Incident Complications in Patients With Type 2 Diabetes in Germany. Diabetes Care. 2018;41(5):971-8.
- 3. Laxy M, Becker J, Kahm K, et al. Utility Decrements Associated With Diabetes and Related Complications: Estimates From a Population-Based Study in Germany. Value Health. 2021;24(2):274-80.
- 4. Alva M, Gray A, Mihaylova B, Clarke P. The effect of diabetes complications on health-related quality of life: the importance of longitudinal data to address patient heterogeneity. Health Econ. 2014;23(4):487-500.
- 5. Lung TW, Hayes AJ, Hayen A, Farmer A, Clarke PM. A meta-analysis of health state valuations for people with diabetes: explaining the variation across methods and implications for economic evaluation. Qual Life Res. 2011;20(10):1669-78.
- 6. Klüsener S, Grigoriev P, Scholz RD, Jdanov DA. Adjusting inter-censal population estimates for Germany 1987-2011: Approaches and impact on demographic indicators. Comparative Population Studies. 2018;43.
- 7. Tamayo T, Brinks R, Hoyer A, Kuß O, Rathmann W. The prevalence and incidence of diabetes in Germany: an analysis of statutory health insurance data on 65 million individuals from the years 2009 and 2010. Deutsches Ärzteblatt International. 2016;113(11):177.
